# Serum proteomic correlates of mental health symptoms in a representative UK population sample

**DOI:** 10.1016/j.bbih.2025.100947

**Published:** 2025-01-15

**Authors:** Anna Dearman, Yanchun Bao, Leonard Schalkwyk, Meena Kumari

**Affiliations:** a Institute for Social and Economic Research, University of Essex, Wivenhoe Park, Colchester, Essex, CO4 3SQ, UK; b School of Mathematics, Statistics and Actuarial Science (SMSAS), University of Essex, Wivenhoe Park, Colchester, Essex, CO4 3SQ, UK; c School of Life Sciences, University of Essex, Wivenhoe Park, Colchester, Essex, CO4 3SQ, UK

**Keywords:** Proteomics, Proximity extension assay, Mental health, Psychological distress, Population health

## Abstract

Poor mental health constitutes a public health crisis due to its high prevalence, unmet need and its mechanistic heterogeneity. A comprehensive understanding of the biological correlates of poor mental health in the population could enhance epidemiological research and eventually help guide treatment strategies. The human bloodstream contains many proteins, several of which have been linked to diagnosed mental health conditions but not to population mental health symptoms, however recent technological advances have made this possible. Here we perform exploratory factor analyses of 184 proteins from two panels (cardiometabolic and neurology-related) measured using proximity extension assays from Understanding Society (the UK Household Longitudinal Study; UKHLS). Data reduction results in 28 factors that explain 55–59% of the variance per panel. We perform multiple linear regressions in up to 5304 participants using two mental health symptom-based outcomes: psychological distress assessed with the general health questionnaire (GHQ-12) and mental health functioning assessed with the 12-Item Short Form Survey, Mental Component Summary (SF12-MCS) using the proteomic factors as explanatory variables and adjusting for demographic covariates. We use backward selection to discard non-significant proteomic factors from the models. Ten factors are independently associated with population mental health symptoms, three of which are immune-related (immunometabolism, immune cell-mediated processes, acute phase processes), three brain-related (neurodevelopment, synaptic processes, neuroprotective processes), two proteolysis-related (proteolysis & the kynurenine pathway, haemostasis & proteolysis), growth factors & muscle, and oxidative stress & the cytoskeleton. Associations partially overlap across the two outcomes, and a sensitivity analysis excluding people taking antidepressants or other central nervous system medications suggestively implicates some of the factors in treatment-resistant poor mental health. Our findings replicate those of case-control studies and expand these to underlie mental health symptomatology in the adult population. More work is needed to understand the direction of causality in these associations.

## Highlights

• Dimensionality reduction yields 28 proteomic factors from 184 serum proteins.
• Ten proteomic factors are independently associated with mental health symptoms.
• Results only partially overlap for different symptom scales: GHQ-12 and SF12-MCS.
• Population mental health has similar biological correlates to psychiatric illness.
• Some results differ when antidepressant users are excluded.

## 1 Introduction

Mental health conditions often emerge during youth (Uhlhaas et al., 2023) through a combination of environmental and biological factors. Together, they create a substantial burden on individuals and societies, and are estimated to contribute 14.6% of years lived with disability across the globe (GBD 2019 Mental Disorders Collaborators, 2022), although the true figure may be substantially larger (Vigo et al., 2016). In England, one in six adults is estimated to have a common mental disorder (McManus et al., 2016). The treatment of mental health conditions remains difficult for several reasons, including their substantial comorbidity and the mechanistic heterogeneity within disorders. Thus, poor mental health constitutes a public health crisis. However, a greater understanding of the biological underpinnings of poor mental health might help to establish biomarkers and suitable treatment approaches (Hill et al., 2024; Le-Niculescu et al., 2021). Furthermore, biological markers of poor mental health can be leveraged in population studies to examine their social and environmental antecedents, which may lead to improved prevention strategies.

Peripheral blood is a convenient tissue source for biomarker testing in humans. Mental health conditions have previously been associated with blood biomarkers of several systems – with much research focussing on inflammatory, immune, metabolic and haemostatic systems – all of whose activities are localised to the blood. Alterations in these systems are adaptive during brief periods of stress: metabolic alterations make energy available for “fight or flight” behaviours while immune, inflammatory and haemostatic alterations prepare the body to cope with any wounds and infections which may be sustained (Austin et al., 2013; Slavich and Irwin, 2014). In contrast to this, chronic stress can lead to maladaptive dysregulation of these systems, resulting in altered levels of their respective biomarkers, which show associations with a range of physical and mental health conditions. Immune markers are implicated in depression (Foley et al., 2023; Majd et al., 2020), bipolar disorder and schizophrenia (Goldsmith et al., 2016), psychosis (Khoury and Nasrallah, 2018), and fear- and anxiety-based disorders (Michopoulos et al., 2017). Immune processes are differentially altered: while inflammation is generally up-regulated in mood disorders and chronic stress, antiviral responses and some cell-mediated processes are down-regulated (Slavich and Irwin, 2014; Maes and Carvalho, 2018). Immune dysregulation causes metabolic dysregulation, which has several mechanistic links with poor mental health (Halaris and Leonard, 2019; Kim et al., 2018; Nousen et al., 2014). Chronic stress and depression have been linked to increases in haemostatic factors (von Känel et al., 2001).

In addition to these processes, associations have been identified between mental health conditions and molecules linked to other biological processes. Several reviews of potential psychiatric biomarkers have highlighted that complement, proteolysis, growth factors, synaptic function, oxidative stress and the cytoskeleton, along with many other biological systems, are implicated in mental health conditions (Comes et al., 2018; García-Gutiérrez et al., 2020; Guest et al., 2016), although the extent to which relevant processes can be detected peripherally may vary; some may take place in the blood, while others may take place in different bodily tissues. In the case of the latter, corresponding proteins may nonetheless be shed into the blood, which functions as a “biological sink” (Huang et al., 2021) potentially rendering them detectable by sensitive analytic methods.

Recent technological advances have enabled the simultaneous measurement of a broad range of low abundance proteins in a sample – “proteomics” – allowing researchers to take a hypothesis-free approach to discover proteins associated with an outcome of interest. This could serve to improve our knowledge of the biological underpinnings of mental health conditions, which in turn may help us find points of intervention and prevention. Large studies are beginning to use proteomic data in combination with genetic data to implicate circulating proteins in a broad range of outcomes, including mental health conditions (Dhindsa et al., 2023; Eldjarn et al., 2023; Zheng et al., 2020). Furthermore, recent proteomic studies of mental health have successfully used continuous symptom measures as outcomes, for example de Sousa Maciel et al. found associations between youth mental health symptoms and markers of immune, haemostatic and neurodevelopmental biology (de Sousa Maciel et al., 2023), and in young adults, Afonin et al. found associations between the general psychopathology factor (“p factor”) and proteins enriched for growth factors and the extracellular matrix (Afonin et al., 2024). Such results suggest that the recent enthusiasm for dimensional models of psychiatric illness, such as HiTOP (Kotov et al., 2017) may be justified. Supporting this view, biomarkers of severe neuropsychiatric conditions such as attention deficit hyperactivity disorder (ADHD), post-traumatic stress disorder (PTSD) and schizophrenia appear to predict broad dimensions of psychopathology in the general population (Hoy et al., 2023).

Much mental health research is based on case-control study designs. However, from a public health perspective, it may be preferable to study representative population samples with minimal ascertainment bias, enabling population-level inference. While there may be a substantial minority of participants with diagnosed mental conditions in such studies, alternative phenotyping strategies are often employed which capture transdiagnostic, continuous measures of mental health. These may more sensitively capture the true burden of poor mental health in the population as some people don't seek treatment for a range of reasons (Salaheddin and Mason, 2016) and some present with symptoms which don't meet diagnostic thresholds. While scales such as GHQ-12 (the General Health Questionnaire) are useful screening tools for common depressive, anxious and somatic disorders (Goldberg et al., 1997) they also seem to capture dimensions of rarer, more severe mental health conditions: using a GHQ-12 threshold to estimate cases of non-psychotic psychiatric morbidity, van Os et al. found that cases showed higher levels of psychotic symptoms compared to non-cases (van Os et al., 1999) and GHQ-12 has some utility in predicting PTSD (Ouimette et al., 2008), while poor quality of life measured by the 12-Item Short Form Survey, Mental Component Summary (SF12-MCS) (Ware et al., 1995) was more likely in individuals with psychosis compared to those without a psychiatric condition (Sanderson and Andrews, 2002) and was significantly worse in those with current PTSD compared to those who had recovered and trauma-exposed PTSD-resistant individuals (Westphal et al., 2011).

To our knowledge, the association of proteins with mental health symptom scales has not been examined in healthy populations across the full adult age range. Here we expand on previous research by examining the association of 184 proteins with two mental health symptom scales in over 5000 participants of a representative population sample: Understanding Society (also known as the UK Household Longitudinal Study; UKHLS). We leverage the correlational nature of the proteome to perform dimensionality reduction, generating proteomic factors for examination rather than individual proteins. Mental health symptoms were assessed during the study's main interview five months prior to blood collection, using two different scales which capture psychological distress and mental health functioning. The use of two different scales allows us to examine the extent to which the biological correlates of these measures overlap. We exploit the richness of Understanding Society to provide associations independent of a variety of covariates not typically accounted for in proteomic analyses.

## 2 Methods

### 2.1 Participants

Understanding Society is a longitudinal household panel survey of the UK with a representative study design (Lynn, 2009). The study began in 2009 and conducts annual interviews to collect a variety of sociodemographic and other information. Data on core topics are collected annually, including mental health symptoms. To reduce interview length, detailed data on other topics are collected less frequently. During waves 2 and 3 (2010–2012), there was a special focus on health and biomarkers. Blood samples were collected during a nurse health assessment which took place in participants’ homes five months after the main annual interview (Institute for Social and Economic Research, 2022a; University of Essex. Institute for Social and Economic Research and National Centre for Social Research, 2022). Blood was sent by post to the processing lab where it was separated into different fractions and frozen within a few days. A subset (n = 6180) of serum samples were sent to Olink® Proteomics (Uppsala, Sweden) for proteomic analysis in 2020.

### 2.2 Symptom measures

Mental health symptoms were measured during the main interview. In the main analysis, we use the measures taken during the main interview which took place approximately five months prior to blood sample collection. In a sensitivity analysis, we examine whether proteomic associations persist using the measures taken at the following main interview, approximately seven months after blood sample collection. Psychological distress was captured with GHQ-12 (the General Health Questionnaire), which has 12 items each scored on a four-point Likert scale running from “not at all” to “much more than usual”. Participants were asked how often they had recently “been able to concentrate”, “lost much sleep over worry”, “been able to overcome difficulties”, “played a useful part in things”, “been capable of making decisions”, “been constantly under strain”, “enjoyed normal day-to-day activities”, “been able to face up to problems”, “felt unhappy or depressed”, “lost confidence”, “thought of yourself as a worthless person”, and “felt reasonably happy” during the last few weeks. Answers to positively valenced questions were inversely coded and the totals added together such that a higher score indicates more psychological distress. Mental health functioning is captured by six mental health related questions included within SF-12 (the 12-Item Short Form Survey). Participants were asked whether “mental health meant they accomplished less”, “mental health meant they worked less carefully”, whether they “felt calm and peaceful”, “had a lot of energy”, “felt downhearted and depressed” and whether “mental health interfered with social life” during the past four weeks. Answers to these items were converted to a single score by the research team and calibrated against population norms to create the mental component summary (SF12-MCS). Prior to analysis, GHQ-12 was reverse-coded to be in line with SF12-MCS, wherein a higher score indicates better wellbeing/functioning. The two scales are highly correlated (Pearson's correlation coefficient = 0.74 in the final analytical sample). Both were centred and scaled for a mean of 0 and a standard deviation of 1 prior to analysis.

### 2.3 Protein measures

Proteins were measured using the proximity extension assay (PEA) developed by Olink® Proteomics (Assarsson et al., 2014). Briefly, a given protein is detected by its binding to two oligonucleotide-linked antibodies, whose resulting proximity enables a quantitative polymerase chain reaction (qPCR) to take place. This yields a C_t_ score that is subsequently adjusted for batch effects and converted to “Normalised Protein eXpression (NPX)” values - an arbitrary unit in the log_2_ scale wherein a one-unit increase represents a doubling in concentration. The background noise for each protein assay is used to calculate a protein-specific limit of detection (LOD). Understanding Society contains measurements of 184 proteins from two of Olink's Target 96 panels: the “cardiometabolic” panel and the “neurology” panel. These panels are designed based on biological processes, disease area, tissue expression and protein class. In the dataset, each protein is broadly normally distributed (Dearman et al., 2023a) and all have similar orders of magnitude, with means ranging from −0.23 to 13.11 and standard deviations ranging from 0.12 to 1.22. To reduce noise in the data associated with poor sample quality, samples were excluded if previously recorded as haemolysed (Guder et al., 2000) or if transit delay (i.e. processing delay) exceeded nine days. Samples with impossible recorded delays (of 0 days or below) were also excluded. Measurements below the LOD were retained as our previous analyses suggest that they do not preclude meaningful associations (Dearman et al., 2023b). All remaining protein measurements were subsequently regressed on transit delay and transit delay squared (i.e. residualised) to correct for its linear and non-linear effects on the abundance of each protein (Dearman et al., 2023b). We conducted exploratory analyses using different dimensionality reduction methods (not published) which indicated that the factor structure within the combined dataset (cardiometabolic and neurology proteins) was difficult to estimate consistently across methods, hence the dataset was split into two: a cardiometabolic dataset and a neurology dataset. For each dataset, individuals with missing protein measurements across the entire panel were excluded. This resulted in sample sizes of 5945 in the cardiometabolic dataset and 5858 in the neurology dataset. Each protein variable was then centred and scaled to give a mean of 0 and a standard deviation of 1, after which any remaining missing measurements were median-imputed (for the cardiometabolic panel 344 values - less than 0.1% - were imputed, and for the neurology panel only five values were imputed). These datasets were then used in the factor analyses. The final sample size was later reduced slightly due to missing outcome and covariate values (see Statistical Analysis).

### 2.4 Covariates and exclusion variables

Participant age, self-reported sex, self-reported ethnicity and educational attainment (highest qualification) were recorded by questionnaire. All participants with proteomic data self-reported as either male or female. Ethnicity and educational attainment are included as covariates in order to identify proteomic factors whose associations with mental health are independent of socially patterned health inequalities. All covariates exhibited bivariate associations with several protein measurements (Dearman et al., 2023b). Age squared was derived from age (to adjust for non-linear effects), after which both variables were centred and scaled for a mean of 0 and a standard deviation of 1. Sex (male, female), ethnicity (White, Asian, Black, Mixed, other ethnicity) and educational attainment (degree, other higher degree, A level etc, GCSE etc, other qualification, no qualification) were treated as categorical variables.

Transit delay was calculated in days using the health assessment date recorded by the nurse and the laboratory receipt date recorded by Fisher BioServices (Bishop's Stortford, UK) who carried out initial processing and storage. Haemolysis was ascertained using light absorbance (Cobas, 2014; Guder et al., 2000) and was recorded by Newcastle upon Tyne Hospitals NHS Foundations Trust (NUTH), who previously carried out a separate battery of biomarker testing (Institute for Social and Economic Research, 2022b).

Participants reported their medication use and this information was subsequently coded according to the British National Formulary (BNF) chapter (British Medical Association, Royal Pharmaceutical Society of Great Britain, 2009). Thus, antidepressants and other central nervous system (CNS) medications were collapsed into one variable.

### 2.5 Statistical analysis

All analyses were carried out using R version 4.2.2.

Factor analysis (FA) was used to characterise the underlying correlational structure within each of the two datasets. FA is preferable over principal component analysis (PCA) because it accounts for measurement error and allows assessment of model fit. However, PCA was performed prior to FA in order to inform the number of factors to be estimated, which is a user-supplied parameter in FA.

An exploratory PCA was performed separately for each panel, using the *prcomp* function from the stats package (v4.2.2) with all default options. The cardiometabolic panel PCA yielded 13 components with an eigenvalue greater than 1 which together explained 65.1% of the variance, whereas the neurology panel PCA yielded 16 components with an eigenvalue greater than 1 which together explained 63.0% of the variance. The number of resulting principal components with an eigenvalue greater than 1 was subsequently used to inform the FA.

FA was then performed using the *fa* function from the psych package (v2.2.9; Revelle, 2022). The default “oblimin” (oblique) rotation was used and the “nfactors” (number of factors) argument was informed as described above. Using this approach, the FA converged successfully for the neurology dataset, but failed to converge after 1000 iterations for the cardiometabolic dataset. Hence, the nfactors argument was reduced from 13 to 12 for the cardiometabolic dataset, after which the FA successfully converged. Default options were used for all other arguments.

The resulting proteomic factors were each centred and scaled for a mean of 0 and a standard deviation of 1 prior to statistical analysis.

In the main analyses, multiple linear regressions were performed using the *glm* function from the stats package (v4.2.2). The outcome variable was either GHQ-12 or SF12-MCS, and the explanatory variables consisted of a proteomic block (i.e. all 28 proteomic factors) and a covariate block (age, age squared, sex, ethnicity, educational attainment). Backward selection was employed, wherein the multiple regression was performed iteratively, each time removing the worst performing proteomic variable (i.e. with the largest p value) until all proteomic variables had a p value less than or equal to a Bonferroni-corrected alpha threshold of 0.025 (0.05 divided by two outcome variables). During each iteration, all covariates were adjusted for, as they were not subject to backward selection. Due to variable missingness, the final sample size was 5304 for GHQ-12 and 5027 for SF12-MCS.

In a *post hoc* analysis, we also checked whether any of the proteomic factors interacted with sex. We used the terms retained in the final multiple regression models described above (i.e. after backward selection was complete) and added a sex interaction term for each retained proteomic factor.

We performed two sensitivity analyses. In sensitivity analysis 1, to examine the impact of the time delay between blood collection and the measurement of mental health symptoms, we repeat the main analysis but instead use measures of GHQ-12 and SF12-MCS taken during the following (rather than the preceding) main interview (around seven months after blood collection). In sensitivity analysis 2, we use the same outcomes as the main analysis (GHQ-12 and SF12-MCS measured at the most recent main interview) but we removed any participants recorded as currently taking CNS medications. The final sample size for sensitivity analysis 1 is 4972 for GHQ-12 and 4973 for SF12-MCS, and the final sample size for sensitivity analysis 2 is 4210 for GHQ-12 and 4030 for SF12-MCS. The process of backward selection was repeated for each sensitivity analysis.

Pearson's correlation coefficients were generated using the *cor* function from the stats package (v4.2.2) to measure correlation between a) the two individual-level outcome variables (GHQ-12 and SF12-MCS) and b) the effect sizes (betas) across outcomes in the main analyses. Welch's two sample t-tests and Pearson's Chi-squared tests were used to compare mental health outcomes, proteomic factors and demographic factors between groups defined by self-reported sex.

### 2.6 Biological descriptions

Due to the relatively small number of proteins measured, enrichment analyses were not performed. Biological annotations, processes and diseases were noted based on a brief literature search.

## 3 Results

### 3.1 Descriptive statistics

Table 1 shows that, based on self-reported sex, there are more women than men in the study. Compared to men, women are slightly younger, have slightly worse mental health, have a significantly different educational profile and show no overall difference in ethnicity profile. Furthermore, all but six proteomic factors exhibit sex differences, with women having significantly higher levels of nine factors and significantly lower levels of thirteen.

**Table 1:** Descriptive statistics. All values are based on the sample used for GHQ-12 analysis, except for SF12-MCS which has slightly more missing values. P values from either T test (continuous variables) or Chi square test (categorical variables) ∗ = p < 0.05, ∗∗ = p < 0.01, ∗∗∗ = p < 0.001, n/s = not significant. Lower scores for GHQ-12 and SF12-MCS indicate more mental health symptoms.

	GHQ-12 sample (main analysis)			
	N (%) or mean (SD)			
	All	Women	Men	
**N**	5304 (100.00)	3212 (60.56)	2092 (39.44)	
**Age**	53.05 (17.31)	52.21 (16.93)	54.34 (17.79)	∗∗∗
**Educational attainment**				∗∗∗
Degree	1161 (21.89)	679 (21.14)	482 (23.04)	
Other higher degree	701 (13.22)	462 (14.38)	239 (11.42)	
A level, etc	1032 (19.46)	524 (16.31)	508 (24.28)	
GCSE, etc	1054 (19.87)	698 (21.73)	356 (17.02)	
Other qualification	565 (10.65)	345 (10.74)	220 (10.52)	
No qualification	791 (14.91)	504 (15.69)	287 (13.72)	
**Ethnicity**				n/s
White	5080 (95.78)	3075 (95.73)	2005 (95.84)	
Asian	118 (2.22)	66 (2.05)	52 (2.49)	
Black	47 (0.89)	30 (0.93)	17 (0.81)	
Mixed	35 (0.66)	23 (0.72)	12 (0.57)	
Other ethnicity	24 (0.45)	18 (0.56)	6 (0.29)	
**Mental health**				
GHQ-12, reverse coded	24.88 (5.43)	24.52 (5.57)	25.44 (5.15)	∗∗∗
SF12-MCS	50.27 (9.44)	49.60 (9.67)	51.29 (8.98)	∗∗∗
**Cardiometabolic factors**				
MR1	−0.007 (0.915)	0.131 (0.906)	−0.219 (0.887)	∗∗∗
MR2	0.015 (0.934)	0.086 (0.886)	−0.093 (0.993)	∗∗∗
MR3	−0.010 (0.878)	0.019 (0.904)	−0.054 (0.833)	∗∗
MR4	−0.011 (0.945)	−0.004 (0.956)	−0.02 (0.927)	n/s
MR5	−0.003 (0.931)	0.058 (0.899)	−0.097 (0.969)	∗∗∗
MR6	−0.006 (0.951)	−0.156 (0.927)	0.224 (0.943)	∗∗∗
MR7	−0.014 (0.954)	−0.250 (0.884)	0.348 (0.944)	∗∗∗
MR8	−0.008 (0.907)	−0.159 (0.862)	0.222 (0.926)	∗∗∗
MR9	−0.007 (0.932)	−0.027 (0.944)	0.023 (0.914)	n/s
MR10	0.005 (0.904)	−0.105 (0.934)	0.174 (0.830)	∗∗∗
MR11	0.001 (0.920)	0.077 (0.898)	−0.116 (0.940)	∗∗∗
MR12	−0.004 (0.930)	−0.058 (0.925)	0.078 (0.933)	∗∗∗
**Neurology factors**				
MR1	−0.012 (0.914)	−0.095 (0.897)	0.116 (0.924)	∗∗∗
MR2	0.013 (0.642)	0.022 (0.712)	0 (0.518)	n/s
MR3	0.006 (0.913)	0.153 (0.877)	−0.22 (0.921)	∗∗∗
MR4	−0.001 (0.885)	−0.228 (0.841)	0.346 (0.838)	∗∗∗
MR5	−0.003 (0.914)	−0.063 (0.895)	0.09 (0.934)	∗∗∗
MR6	−0.009 (0.881)	0.209 (0.790)	−0.344 (0.907)	∗∗∗
MR7	−0.008 (0.887)	0.028 (0.902)	−0.064 (0.86)	∗∗∗
MR8	0.005 (0.881)	−0.042 (0.880)	0.076 (0.878)	∗∗∗
MR9	−0.004 (0.867)	−0.134 (0.857)	0.195 (0.845)	∗∗∗
MR10	−0.008 (0.868)	−0.243 (0.834)	0.353 (0.793)	∗∗∗
MR11	−0.005 (0.874)	−0.018 (0.886)	0.016 (0.856)	n/s
MR12	−0.009 (0.845)	−0.078 (0.856)	0.097 (0.816)	∗∗∗
MR13	−0.013 (0.905)	0.000 (0.900)	−0.033 (0.911)	n/s
MR14	−0.004 (0.903)	−0.005 (0.917)	−0.002 (0.881)	n/s
MR15	−0.003 (0.898)	−0.284 (0.809)	0.429 (0.857)	∗∗∗
MR16	0.002 (0.917)	0.077 (0.975)	−0.113 (0.806)	∗∗∗

### 3.2 Factor analysis

Table 2 shows key features of the 12 factors observed within the cardiometabolic panel and 16 factors from the neurology panel. These factors are labelled using the default names assigned to them by the software, i.e. MR1 to MR12 for the cardiometabolic panel and MR1 to MR16 for the neurology panel. We additionally specify the factor's corresponding panel throughout. Together, the 12 cardiometabolic factors explained 58.7% of the variance and the 16 neurology factors explained 54.9% of the variance for their respective panel datasets. Model fit was good (see Table S1 in supplementary materials). Top eigenvalues were 5.3 and 4.9 for the cardiometabolic and neurology panels, respectively (see scree plots in supplementary materials Fig. S1). The top proteins – those with the strongest loadings - are also shown in Table 2.

**Table 2:** Proteomic factors arranged by panel and by eigenvalue.

Factor	Eigen value	Variance Explained Per Panel (%)	Cumulative Variance Explained Per Panel (%)	Top proteins^†^ (loading)
Cardiometabolic				
MR12	5.30	5.76	5.76	ST6GAL1 (0.624), ANG (0.598), CCL18 (0.548), CCL5 (0.525), GNLY (0.473), CFHR5 (0.459), COL18A1 (0.444), CCL14 (0.424), F11 (0.314)
MR9	5.24	5.70	11.46	THBS4 (0.708), COMP (0.698), PAM (0.438), EFEMP1 (0.418), GAS6 (0.396), CRTAC1 (0.358, LYVE1 (0.345), TGFBR3 (0.334)
MR5	5.13	5.57	17.03	SELL (0.740), CR2 (0.690), PTPRS (0.400), KIT (0.395), VCAM1 (0.392), NOTCH1 (0.349), TIE1 (0.325), LYVE1 (0.321), FCGR3B (0.303)
MR7	5.11	5.56	22.58	IGFBP6 (0.769), CST3 (0.629), REG1A (0.487), COL18A1 (0.428), PCOLCE (0.400), CD59 (0.393), TGFBR3 (0.354), VASN (0.353), CCL14 (0.340), MFAP5 (0.336), EFEMP1 (0.334), CDH1 (0.328), PRSS2 (0.320), UMOD (−0.333)
MR2	5.11	5.56	28.14	SERPINA5 (0.638), PROC (0.606), CNDP1 (0.602), F11 (0.518), IGFBP3 (0.508), F7 (0.501), APOM (0.463), FETUB (0.424), FCN2 (0.397), SAA4 (0.365), MEGF9 (0.351)
MR4	4.45	4.84	32.98	DEFA1 (0.891), ITGAM (0.858), LCN2 (0.747), CA4 (0.597), QPCT (0.386)
MR1	4.19	4.55	37.53	ANGPTL3 (0.446), ICAM1 (0.407), OSMR (0.399), LILRB1 (0.398), VCAM1 (0.353), LILRB2 (0.349), TIMD4 (0.344 )
MR3	4.14	4.49	42.03	REG3A (0.737), PLTP (0.639), LTBP2 (0.578), FAP (0.507), MFAP5 (0.488), MET (0.404), PLA2G7 (0.394), PTPRS (0.368), UMOD (0.367), PLXNB2 (0.311)
MR11	4.12	4.48	46.51	GP1BA (0.563), CD46 (0.478), TIMP1 (0.430), NID1 (0.420)
MR8	4.08	4.44	50.95	CES1 (0.532), TGFBI (0.474), PRCP (0.419), C1QTNF1 (0.419), PLXNB2 (0.393), MEGF9 (0.370)
MR6	3.72	4.04	54.99	CA1 (0.913), CA3 (0.786), SOD1 (0.759), CD59 (0.340)
MR10	3.41	3.71	58.70	APOM (0.437), NCAM1 (0.433), DPP4 (0.398), AOC3 (0.360), CHL1 (0.358), ENG (0.337)
Neurology				
MR16	4.91	5.34	5.34	ROBO2 (0.593), CNTN5 (0.534), NTRK2 (0.491), NTRK3 (0.467), SCARF2 (0.464), PRTG (0.444), CDH6 (0.397), GDNFR-ALPHA-3 (0.388), MDGA1 (0.369), FLRT2 (0.335), DDR1 (0.315), TMPRSS5 (0.313)
MR7	4.57	4.97	10.31	CRTAM (0.667), GZMA (0.620), IL12 (0.586), FCRL2 (0.426), DKK-4 (0.370)
MR1	4.00	4.35	14.66	EDA2R (0.449), JAM-B (0.410), SCARA5 (0.402), VWC2 (0.389), SMOC2 (0.370), SCARB2 (0.360), ADAM-22 (0.347)
MR13	3.71	4.03	18.69	CDH3 (0.704), EPHB6 (0.477), ADAM-22 (0.303), N2DL-2 (0.301)
MR2	3.62	3.93	22.63	MANF (0.815), NRP2 (0.693), CLEC1B (0.557), NR-CAM (0.411), CTSS (0.394), CPM (0.345), TNFRSF21 (0.329)
MR11	3.30	3.59	26.22	SIGLEC1 (0.550), MSR1 (0.439), SIGLEC-9 (0.311)
MR15	3.10	3.37	29.58	GDF-8 (0.742), RGMA (0.569), RGMB (0.446), BMP-4 (0.342)
MR9	3.04	3.31	32.89	SMPD1 (0.744), NAAA (0.656), CTSC (0.353)
MR3	2.90	3.15	36.04	BCAN (0.807), NCAN (0.736), CADM3 (0.397), SPOCK1 (0.365)
MR8	2.74	2.98	39.02	LAT (0.738), PLXNB3 (0.720), GPC5 (0.462), GAL-8 (0.316)
MR4	2.73	2.97	41.99	NEP (0.630), KYNU (0.557), CPM (0.456), PVR (0.446)
MR14	2.67	2.90	44.89	NMNAT1 (0.701), EZR (0.589), CTSC (0.423), CTSS (0.419), CD38 (0.351)
MR12	2.55	2.77	47.67	MAPT (0.544), BETA-NGF (0.378), CLEC1B (−0.297)
MR10	2.46	2.68	50.34	MATN3 (0.456), RSPO1 (0.377), CD38 (0.322), SFRP-3 (0.304), N-CDASE (−0.352)
MR5	2.35	2.56	52.90	HAGH (0.843), LXN (0.708), EZR (0.259)
MR6	1.84	2.00	54.90	LAYN (0.314), SFRP-3 (−0.360), WFIKKN1 (−0.610)

† Top proteins includes either: all of the proteins with an absolute loading greater than 0.3 or, where this constitutes less than three proteins, the three with the biggest absolute loadings.

Most of the top proteins have positive loadings, so unless otherwise stated, a negative association between a factor and an outcome indicates that a high level of wellbeing/functioning is associated with lower levels of the contributing proteins.

### 3.3 Associations with mental health symptoms

**Fig. 1:**
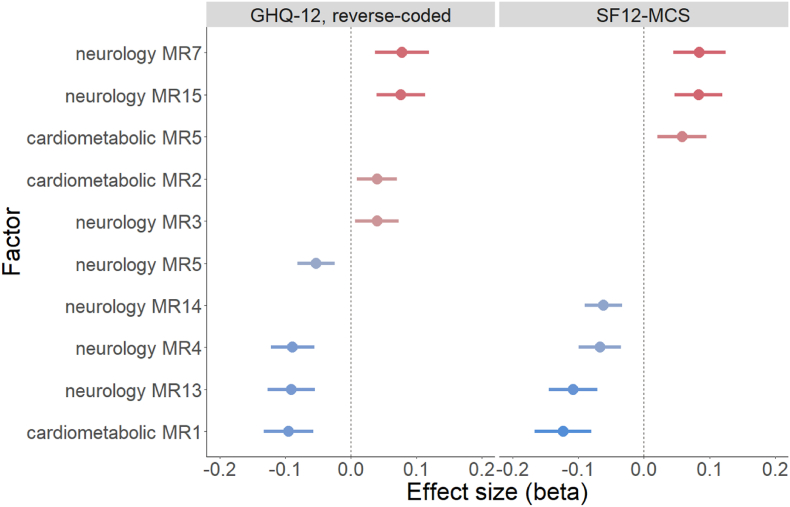
Forest plots for proteomic factors significantly associated with one or both measures of mental health symptoms in a multiple regression. Lower scores for GHQ-12 and SF12-MCS indicate more mental health symptoms.

After backward selection, the model for GHQ-12 retained eight proteomic factors and the model for SF12-MCS retained seven (see Table 3). A total of ten factors are retained in at least one of the two models, with eighteen being discarded in both. Five factors are retained in both models, a further three are associated with GHQ-12 only, and two others are associated with SF12-MCS only, totalling fifteen associations, eight (53.3%) of which are negative. For each of the five factors associated with both outcomes, the direction of effect is concordant, with a Pearson's correlation coefficient across betas of 0.983. Effects are generally larger for SF12-MCS than for GHQ-12, with absolute betas ranging from 0.058 to 0.123 for the former (mean 0.084, median 0.083) and 0.040–0.094 (mean 0.070, median 0.077) for the latter. See Fig. 1 for forest plots in which factors are presented in order of their respective beta coefficients.

**Table 3:** Multiple regression estimates for each factor in the main analysis and the sensitivity analysis. ∗ = p < 0.025, ∗∗ = p < 0.005, ∗∗∗ = p < 0.0005. Lower scores for GHQ-12 and SF12-MCS indicate more mental health symptoms. Backward selection was used to identify parsimonious models, thus missing estimates signify that the factor was dropped from the model. AD = antidepressant.

			GHQ-12 (reverse coded) Beta		SF12-MCS Beta	
Factor	Top three proteins	Possible biological process(es)	Main analysis	Excluding AD users	Main analysis	Excluding AD users
Cardiometabolic						
MR5	SELL, CR2, PTPRS	acute phase processes		0.055 ∗	0.058 ∗∗	
MR2	SERPINA5, PROC, CNDP1	haemostasis & proteolysis	0.040 ∗			
MR1	ANGPTL3, ICAM1, OSMR	immunometabolism	−0.094 ∗∗∗	−0.100 ∗∗∗	−0.123 ∗∗∗	−0.066 ∗∗
Neurology						
MR7	CRTAM, GZMA, IL12	immune cell-mediated processes	0.078 ∗∗∗	0.063 ∗∗	0.084 ∗∗∗	0.076 ∗∗∗
MR13	CDH3, EPHB6, ADAM-22	neurodevelopment	−0.091 ∗∗∗	−0.062 ∗∗	−0.108 ∗∗∗	−0.077 ∗∗∗
MR15	GDF-8, RGMA, RGMB	growth factors & muscle	0.077 ∗∗∗		0.083 ∗∗∗	0.058 ∗∗
MR3	BCAN, NCAN, CADM3	synaptic processes	0.040 ∗			
MR4	NEP, KYNU, CPM	proteolysis & the kynurenine pathway	−0.089 ∗∗∗		−0.067 ∗∗∗	
MR14	NMNAT1, EZR, CTSC	neuroprotective processes		−0.046 ∗∗	−0.062 ∗∗∗	−0.074 ∗∗∗
MR5	HAGH, LXN, EZR	oxidative stress & the cytoskeleton	−0.053 ∗∗∗

Five factors are associated with both mental health measures. Cardiometabolic MR1 has the largest absolute association with both GHQ-12 (beta = −0.094, p = 9.44e-07) and SF12-MCS (beta = −0.123, p = 2.46e-08). Neurology MR13 has the second largest absolute association with both GHQ-12 (beta −0.091, p = 1.03e-06) and SF12-MCS (beta = −0.108, p = 1.07e-08). Neurology MR7 has the largest positive association with GHQ-12 (beta = 0.078, p = 1.8e-04) and with SF12-MCS (beta = 0.084, p = 3.22e-05). Also associated with both outcomes are neurology MR15 (GHQ-12 beta = 0.077, p = 5.02e-05, SF12-MCS beta = 0.083, p = 8.73e-06) and neurology MR4 (GHQ-12 beta = −0.089, p = 1.68e-07, SF12-MCS beta = −0.067, p = 5.35e-05).

Three factors were only associated with GHQ-12: neurology MR5 (GHQ-12 beta = −0.053, p = 2.58e-04), cardiometabolic MR2 (beta = 0.040, p = 0.010) and neurology MR3 (beta = 0.040, p = 0.018). Two factors were only associated with SF12-MCS: neurology MR14 (beta = −0.062, p = 2.03e-05) and cardiometabolic MR5 (beta = 0.058, p = 0.002).

A *post hoc* analysis checking for sex interactions across the factors revealed a nominally significant interaction with sex for cardiometabolic MR1 only (GHQ-12 beta = −0.075, p = 0.040, SF12-MCS beta = −0.075, p = 0.042, reference category female). For a scatter plot, see supplementary materials Fig. S2.

The main analysis examines proteomic associations with mental health outcomes measured five months prior to the blood collection, which are the most synchronous measures available. We performed a sensitivity analysis using the same mental health outcomes measured seven months after blood collection. Of the ten factors identified in the main analysis, six are recapitulated (cardiometabolic MR1 and MR2; neurology MR4, MR7, MR13 and MR15) and a further two are significant (cardiometabolic MR6 and neurology MR6), eight in total. Unlike in the main analyses, the significant proteomic factors are identical for the two outcomes. For each proteomic factor, coefficient signs are concordant across outcome measures and time points.

We performed a second sensitivity analysis restricted to those not taking CNS medications (including antidepressants). After backward selection, five proteomic factors were retained in the final models for both outcomes (see Table 3), with none of the eighteen previously discarded factors becoming significant. Of the five factors associated with both outcomes in the main analysis, three remained robustly associated with both (cardiometabolic MR1, neurology MR7 and neurology MR13) while neurology MR15 lost its association with GHQ-12 and neurology MR4 lost both of its associations. All of the three factors uniquely associated with GHQ-12 in the main analysis lost their associations (cardiometabolic MR2, neurology MR3 and neurology MR5). Both of the factors uniquely associated with SF12-MCS in the main analysis gained an association with GHQ-12 but neurology MR14 retained its association with SF12-MCS whereas this association was lost for cardiometabolic MR5.

## 4 Discussion

In an analysis of protein factors and their associations with recently-measured mental health measurements, we find ten factors associated with mental health, five of which are associated with both GHQ-12 and SF12-MCS, three others of which are associated with GHQ-12 only and the remaining two with SF12-MCS only. Associations were robust to a variety of sociodemographic factors not commonly accounted for in proteomic studies. Thus, we suggest that there are common and distinct biological factors that underpin psychological distress and mental health functioning. Some associations varied in a sensitivity analysis which excluded everybody who was taking antidepressants and other CNS medications.

A range of biological processes have previously been linked to mental health conditions. A review of some of the intersecting biological processes which underly poor mental health identified disruptions in inflammation, metabolism, the kynurenine pathway, neuroprotection and oxidative stress (Halaris and Leonard, 2019). Many of these processes have been recapitulated in papers on potential psychiatric biomarkers, which additionally implicate complement, haemostasis, proteolysis, the adaptive immune system, neurodevelopment, growth factors, synaptic function and the cytoskeleton, along with other biological processes (Comes et al., 2018; de Sousa Maciel et al., 2023; García-Gutiérrez et al., 2020; Guest et al., 2016). Several reviews and perspectives have been published which outline the interplay between many of these biological processes in the context of stress and mental health conditions (e.g. Branchi et al., 2021; Halaris and Leonard, 2019; Maes and Carvalho, 2018; Slavich and Irwin, 2014; Ulbrich et al., 2021). It is important to examine mental health using transdiagnostic, continuous measures of symptoms in a representative population sample. Such research can identify the social and environmental antecedents of poor mental health and enable population-level inferences independently of health service use. Our findings extend previous observations of the biological correlates of poor mental health to recently-measured mental health symptoms in the population. It is therefore noteworthy that many of the proteins which were associated with non-clinical measures of mental health symptoms in the present study have been previously linked with severe mental health conditions, such as schizophrenia (Smirnova et al., 2019) and PTSD (Waszczuk et al., 2023). This suggests that the biological underpinnings of milder, more common manifestations of poor mental health may be shared with those of severe illness – a notion that is supported by some studies identifying associations between genetic biomarkers of psychiatric disease risk and continuous measures of sub-clinical symptoms, behaviours and neurobiological phenotypes in healthy or general population samples (e.g. Liu et al., 2023; Pries et al., 2020; Schlag et al., 2022).

Due to the selective nature and limited number of proteins measured in the current study (92 cardiometabolic and 92 neurology-related proteins), characterisation of the factors using objective enrichment analyses is not plausible. Furthermore, interpretation of the factors is complicated by the proteins’ tendencies to have multiple biological roles, to exhibit multiple disease associations and to be expressed with varying tissue specificity. Hence, the following section is a non-exhaustive labelling of the significant factors according to the biological processes they might represent by identifying commonalities in their most strongly loading proteins.

**Cardiometabolic MR5** could represent “acute phase processes”, including inflammation and complement. Its top protein, SELL (a.k.a. L-selectin), helps facilitate the entry of immune cells into tissues during inflammation (Bernimoulin et al., 2003; Ivetic et al., 2019), while its third protein, PTPRS (a.k.a. RPTPσ), has been linked with inflammatory regulation (Bunin et al., 2015). Its second protein, CR2, is part of the complement system, which is implicated in schizophrenia (Woo et al., 2020). **Cardiometabolic MR1** could represent “immunometabolism” as its three most strongly-loading proteins (ANGPTL3, ICAM1 and OSMR) have all been linked with inflammation (Bui et al., 2020; West et al., 2017; Zhang et al., 2023) which is driven by the innate immune system. All three proteins are also linked with metabolic health via lipid biology: ANGPTL3 is well known for its role in lipid metabolism, and oncostatin M (the ligand for OSMR) has been shown to promote ICAM1 expression in adipocytes (fat cells) (Stephens et al., 2018). ICAM1 and OSMR have been linked to depressive episodes in a recent study (Eldjarn et al., 2023). The associations of cardiometabolic MR5 and MR1 (which both index inflammatory processes) with mental health accord with a vast literature that links inflammatory processes and mental health and serve to provide new insight into the processes by which these links might occur. We found a significant sex interaction such that a stronger link between mental health and cardiometabolic MR1 (immunometabolism) was apparent for men compared to women (see supplementary materials Fig. S2), which contradicts previous studies which find links between mood disorders and BMI/obesity in women but not men (Carpenter et al., 2000; Stunkard et al., 2003). The reason for this is unclear.

**Neurology MR4** could be capturing “proteolysis” as its three most strongly loading proteins (NEP a.k.a. membrane metalloendopeptidase, KYNU a.k.a. kynureninase, and CPM) all degrade proteins or their products (peptides or amino acid metabolites) (Bayes-Genis et al., 2016; Deiteren et al., 2009; Phillips, 2014). Proteolytic proteins such as the matrix metalloproteinases are involved in extracellular matrix turnover in the CNS (Ulbrich et al., 2021) and have been implicated in mental health conditions (Cathomas et al., 2024; Comes et al., 2018). The presence of KYNU in this list also implicates the kynurenine pathway, which is strongly linked to mental health symptoms, episodes and conditions (Arnau-Soler et al., 2019; Branchi et al., 2021; Eldjarn et al., 2023; Skorobogatov et al., 2023).

**Neurology MR14** could be capturing “neuroprotective processes”. Its top three proteins include EZR (also a top protein for neurology MR5), NMNAT1 and CTSC, all of which may be neuroprotective (Niemeyer et al., 2020; Sasaki et al., 2016; Vega et al., 2018). Neuroprotective mechanisms have been identified as key contributors in the progression of mental health conditions (Halaris and Leonard, 2019).

**Neurology MR5** could be capturing “oxidative stress and the cytoskeleton”. All of its top three proteins (HAGH, LXN, EZR) were strongly associated with haemolysis (burst red blood cells) in previous analyses (Dearman et al., 2023b) and, in red blood cells, cell shape is sometimes linked to oxidative stress (Ruggeri et al., 2018). Accordingly, two of this factor's top proteins have been linked to cell shape via their role in the cytoskeleton: LXN (He et al., 2021) and EZR (Grune et al., 2002) and two (HAGH and EZR) have been linked to oxidative stress (Ahmad et al., 2020; Grune et al., 2002; Kim et al., 2010). Oxidative stress has been linked to several mental health conditions (Comes et al., 2018), and abuse (Kurkinen et al., 2023) while schizophrenia has been linked to both oxidative stress and the cytoskeleton (Jiang et al., 2019). Reactive oxygen species may play a role in PTSD via their inhibitory effects on GABA-ergic signalling which normally serves to inhibit the amygdala during fear extinction, and also via their effects on the inflammatory response (Preston et al., 2018). However, it is possible that this factor captures pre-analytic factors (Dearman et al., 2023b) although the mechanism by which these would associate with mental health symptoms is unclear.

**Cardiometabolic MR2** appears to capture “haemostasis” via two of its top three proteins (PROC a.k.a. protein C and SERPINA5 a.k.a. protein C inhibitor), although, like neurology MR4, it also appears to capture “proteolysis”. The anti-proteolytic activity of the serine protease inhibitor SERPINA5 is leveraged to promote clotting (Elisen et al., 1998; Yang and Geiger, 2017) by inhibiting PROC which degrades clotting factors (Dahlbäck and Villoutreix, 2005). Haemostatic processes are closely linked to complement (Oncul and Afshar-Kharghan, 2020). The factor's third protein, CNDP1 (a.k.a. serum carnosinase), also breaks down proteins (Boldyrev et al., 2013). Both SERPINA5 and CNDP1 have been linked to schizophrenia (Smirnova et al., 2019) as have other serine protease inhibitors (Comes et al., 2018).

**Neurology MR7** may be capturing “immune cell-mediated processes” as its three most strongly-loading proteins (CRTAM, GZMA and IL12), are all related to natural killer (NK) cell and cytotoxic T lymphocyte (CTL) processes (Arase et al., 2005; Pardo et al., 2002; Park et al., 2003). This factor's association with mental health accords with previous findings that aspects of cell-mediated immunity such as natural killer cell activity are altered during depressive episodes (Maes and Carvalho, 2018) and may support the monocyte-T-lymphocyte hypothesis of major depression (Maes et al., 1995).

**Neurology MR13** may be capturing “neurodevelopment” as its three most strongly-loading proteins (CDH3 a.k.a cadherin 3, EPHB6 and ADAM-22) have all been linked to neurodevelopmental processes: CDH3 in visual system development (Osterhout et al., 2014) and EPHB6 and ADAM-22 in brain development (He et al., 2023; Hsia et al., 2019). All three proteins have been implicated in autism (Al-Mazidi et al., 2023; Butler et al., 2015; Fang et al., 2021) which is a neurodevelopmental condition associated with increased risk of co-occurring mental health diagnoses (Lai et al., 2019). A different member of the cadherin family has recently been linked to mental health symptoms in a plasma proteomic study of youth (de Sousa Maciel et al., 2023).

**Neurology MR15** could be capturing “growth factors” as its three most strongly-loading proteins (GDF-8 a.k.a. myostatin, RGMA and RGMB) all play an inhibitory role in development (Jiang et al., 2004; Siebold et al., 2017). This accords with previous findings that circulating growth factors have been linked to mental health outcomes (Afonin et al., 2024; Comes et al., 2018; Guest et al., 2016). However, this factor could also be capturing “muscle biology”: while RGMA and RGMB are widely expressed, GDF-8 (a.k.a. myostatin) is mostly expressed in muscle and may be an indicator of skeletal muscle mass and function (for review, see Baczek et al., 2020). Muscle has plausible links to brain health via numerous pathways, including via myokines which promote neurogenesis by enhancing brain-derived neurotrophic factor (BDNF) production and by increasing kynurenine aminotransferase enzymes which reduce kynurenine levels (Pedersen, 2019).

**Neurology MR3** could be capturing “synaptic processes”. Its top three proteins (BCAN, NCAN, CADM3) are all highly expressed in the brain and have been associated with the synapse (Blosa et al., 2015; Peters van Ton et al., 2020; Song et al., 2022). BCAN and NCAN are found in perineuronal nets (PNNs) which are found in the extracellular matrix, and which play a role in synaptic plasticity, memory and psychiatric illnesses (Fawcett et al., 2019). They have both been associated with symptom severity in PTSD (Waszczuk et al., 2023). A genetic study implicated NCAN in bipolar disorder and schizophrenia (Mühleisen et al., 2012). Synaptic transmission and function have been implicated in reviews of potential psychiatric biomarkers (Comes et al., 2018; García-Gutiérrez et al., 2020). More generally, extracellular matrix biology has been implicated in mental health (Dityatev et al., 2021), including in a genome-wide gene-environment interaction study (along with synaptic plasticity; Wendt et al., 2021) and in a recent proteomics study of young adults (Afonin et al., 2024).

The measurement of two mental health symptom scales yields intriguing results that warrant further consideration. Firstly, the effect size estimates are generally of a greater magnitude for mental health functioning (SF12-MCS) than for psychological distress (GHQ-12). This may suggest that the biological correlates of the former are more easily measured in the blood. Secondly, while most of the biological correlates for each outcome are shared (immunometabolism, immune cell-mediated processes, neurodevelopment, growth factors and proteolysis and the kynurenine pathway), a substantial proportion is not shared, shedding light on the differences between the two measures: GHQ-12 is uniquely linked to three factors capturing haemostasis & proteolysis, synaptic processes, and oxidative stress & the cytoskeleton, while SF12-MCS is uniquely linked to acute phase processes and neuroprotective processes.

Asynchronous measurement of symptoms and proteins entails measurement error, as it is likely that most participants’ symptoms improved or worsened to a greater or lesser extent by the time blood was collected. As a result, this study could have failed to detect any acute, transient changes in peripheral biology which might correlate with poor mental health, and the magnitude of coefficients may be underestimated. Nevertheless, the fact that our findings appear to replicate those from studies which differed in terms of design, sample demographics, mental health outcomes and biological assay suggests that our measures are sufficiently contemporaneous for an exploratory analysis of the biological underpinnings of poor mental health and that the time delay does not preclude the detection of associations consistent with previous literature. We speculate that the symptoms, their biological correlates, or both, are enduring enough in the sample to explain our findings.

In the sensitivity analysis which excluded individuals taking antidepressants (and other CNS medications), five factors lost their associations with GHQ-12 (haemostasis & proteolysis, synaptic processes, proteolysis & the kynurenine pathway, growth factors, and oxidative stress & the cytoskeleton). We have previously identified an association between GHQ-12 and the inflammatory marker C-reactive protein (CRP) in users of antidepressants but not non-users in Understanding Society (Hughes and Kumari, 2017), consistent with the well-established notion of an inflammatory, treatment-resistant subtype of depression. We speculate that, in our analyses, proteomic factors whose associations with GHQ-12 are lost after exclusion of antidepressant users are indicative of additional biological processes which underpin treatment-resistant poor mental health. For example, it is plausible that neurology MR5 (oxidative stress & the cytoskeleton) is linked to treatment-resistant poor mental health, as some antidepressants could exacerbate mitochondrial dysfunction (resulting in increased oxidative stress), thus perpetuating poor mental health (Preston et al., 2018). The fact that, in contrast to the previous observation for CRP, our immunometabolic and immune cell-mediated factors are associated with mental health in non-users highlights the multi-faceted nature of the immune response in depression.

### 4.1 Advantages

This study has several strengths. These analyses were conducted in a large population study representing the entire adult age range. More than one measure of mental health symptoms was examined, which showed overlapping and distinct associations with proteomic factors, some of which altered in an analysis restricted to individuals untreated by CNS medications. We adjusted for sociodemographic factors which are not typically accounted for in proteomic studies.

The proteins apparently serve to index processes for which they were not explicitly made commercially available, nor may they have been the best proxy. Nevertheless, leveraging the correlational nature of these proteins in a factor analysis has enabled us to describe associations with mental health which replicate previous case-control studies. As such, we demonstrate the feasibility of utilising proteomic measurements in large population samples to measure latent biological processes with established links to mental health, and their social and environmental causes.

### 4.2 Limitations and recommendations for future work

The study did not comprehensively examine the serum proteome as it relied on panels which constitute 184 proteins which are highly enriched for cardiometabolic and neurology-related processes.

While model fit was good in the factor analyses, only 55–59% of the variance in each panel was explained which leaves a substantial amount unexplained. However, this may be in line with other studies, for example a study which examined similar Olink® neurology proteins in the Lothian Birth Cohorts and INTERVAL studies found that 63–74% of the variance was explained by the first 17 components using PCA (Harris et al., 2020).

As discussed above, despite the similarity between our observations and those in case-control studies of psychiatric biomarkers, in our study mental health symptoms were measured five months prior to blood collection which may limit our ability to detect any acute or transient associations with proteins, and to accurately estimate the magnitudes of associations. Some factors were robustly associated when symptoms were instead measured seven months after blood collection – this and the fact that other associations were lost warrants further investigation.

In the present study, analyses are approximately cross sectional and therefore we cannot discount reverse causation or residual confounding. Both mental health and the circulating proteome are likely to be affected by factors such as medications, physical health including recent infection, and health behaviours such as smoking (Plurphanswat et al., 2017), which were not accounted for in this study, and which could act as mediators or confounders. For example, contraceptive use has been associated with a substantial amount of variance in the plasma proteome (Dordevic et al., 2023) and a range of mental health conditions have been associated with deviations in biomarker-based indices of physiological functioning across multiple organ systems (Tian et al., 2023), implicating physical health as an important factor. Thus, more work is required to estimate the relationships between these factors.

Longitudinal approaches could support the inference of causal pathways. It is plausible that some proteomic factors may reflect pre-existing stable risk factors, for example neurology MR13 appears to capture neurodevelopment, and all three of its top proteins have been previously linked to autism, a neurodevelopmental condition which entails elevated risk of poor mental health (Lai et al., 2019). It is plausible that other factors exhibit a bidirectional relationship with mental health symptoms, for example cardiometabolic MR1 (immunometabolism) which may reflect metabolic disruption (Pan et al., 2012). Furthermore, mental health symptoms could be used to construct long term mental health trajectories, the proteomic correlates of which could then be examined. Such a prospective approach could contribute to burgeoning research efforts aimed at predicting mental health outcomes from biomarkers, for example circulating biomarkers have been linked to future psychosis-like experiences and transitions to psychosis in young people (Hernandez et al., 2023; Taylor et al., 2020). Our first sensitivity analysis found that, of eight proteomic factors prospectively associated with mental health, only six were also associated with recent mental health, suggesting that the remaining two may hold predictive value for future poor mental health. However, differences between the main and sensitivity analyses could be due to loss-to-follow-up which impacts sample size and could feasibly alter relevant characteristics of the sample.

As mental health conditions are notoriously heterogeneous, future work could focus on subtyping individuals according to symptom profiles based on the individual GHQ-12 or SC12-MCS items.

It is possible that the loss of associations between GHQ-12 and five factors in sensitivity analysis 2 is reflective of reduced power rather than something biologically meaningful. This and our unexpected findings represent opportunities for replication in other population datasets.

## 5 Conclusion

Here we perform an exploratory analysis of the association of proteins with mental health symptoms in the adult population. Using factor analysis, we describe a number of biological constructs relevant to mental health. Our findings accord with previous studies of psychiatric illness and stress, whose biological correlates include inflammatory, haemostatic and neuroprotective factors. There was partial overlap between the factors associated with two symptom scales: psychological distress and mental health functioning, with each exhibiting some distinct associations. Further studies are needed to examine the mechanisms that underpin these associations. Associations which weren't robust to the exclusion of antidepressant users may shed light on the biological underpinnings of treatment-resistant poor mental health.

## CRediT authorship contribution statement

**Anna Dearman:** Writing – review & editing, Writing – original draft, Formal analysis. **Yanchun Bao:** Writing – review & editing, Methodology. **Leonard Schalkwyk:** Writing – review & editing. **Meena Kumari:** Writing – review & editing, Supervision, Project administration, Methodology, Funding acquisition, Conceptualization.

## Ethical statement

All methods were carried out in accordance with Information Commissioner's Office guidelines and regulations. Informed consent was obtained from all participants. The University of Essex Ethics Committee approved data collection on Understanding Society main study. Approval for asking for consent and the collection of biosocial data by trained nurses was obtained from the National Research Ethics Service (Understanding Society—UK Household Longitudinal Study: A Biosocial Component, Oxfordshire A REC, Reference: 10/H0604/2).

## Declaration of competing interest

The authors declare that they have no known competing financial interests or personal relationships that could have appeared to influence the work reported in this paper.

## Data Availability

The research data are distributed by the UK Data Service. DOI: http://doi.org/10.5255/UKDA-Series-2000053.
