# Supplementary material for: Serum proteomic correlates of mental health symptoms in a representative UK population sample
Source: Brain Behav Immun Health. 2025 Jan 15;44:100947. doi: 10.1016/j.bbih.2025.100947 (PMC11795072; doi:10.1016/j.bbih.2025.100947)
Supplement: Multimedia component 1 [file mmc1.docx]

# Supplementary materials

| **Metric** | **Cardiometabolic** | **Neurology** |
| --- | --- | --- |
| p value (If n.obs > 0, then what is the probability of observing a Chi-square (based on Bartlett’s test) this large or larger?) | 0 | 0 |
| fit (how well does the factor model reproduce the correlation matrix?) | 0.983 | 0.971 |
| fit.off (how well are the off diagonal elements reproduced?) | 0.997 | 0.998 |
| RMSR (root mean square of the residuals) | 0.02 | 0.01 |
| TLI (factoring reliability) | 0.869 | 0.916 |
| RMSEA index | 0.047 (90% CI: 0.047, 0.048) | 0.035 (90% CI: 0.034, 0.035) |

Table S1. Model fit statistics for factor analyses of each proteomic panel


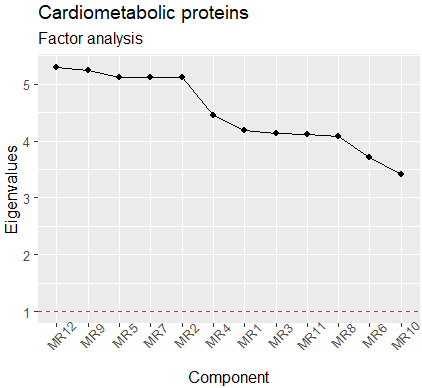

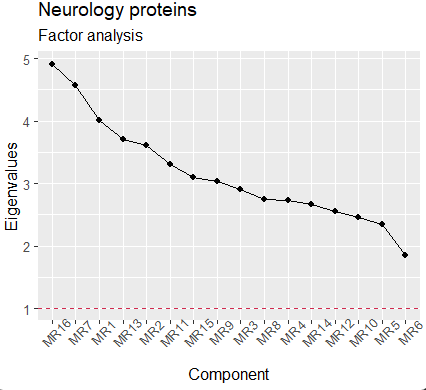


1. b.

Figure S1. Factor analysis scree plots. a. Cardiometabolic panel, b. Neurology panel


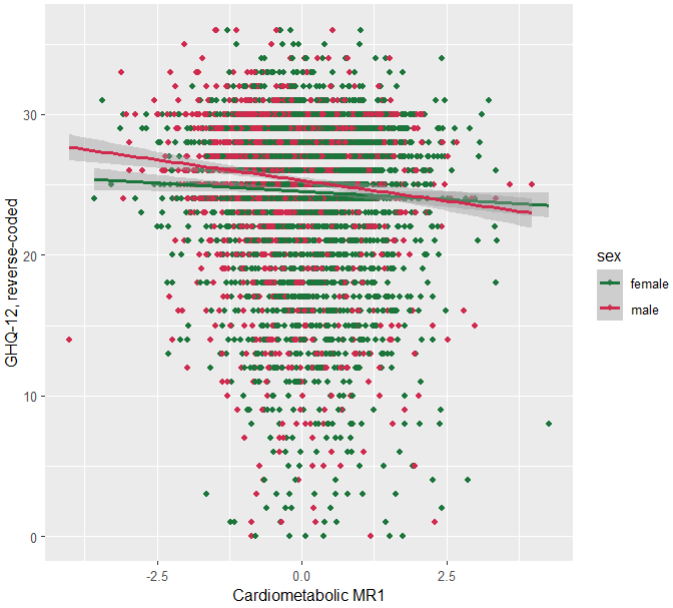

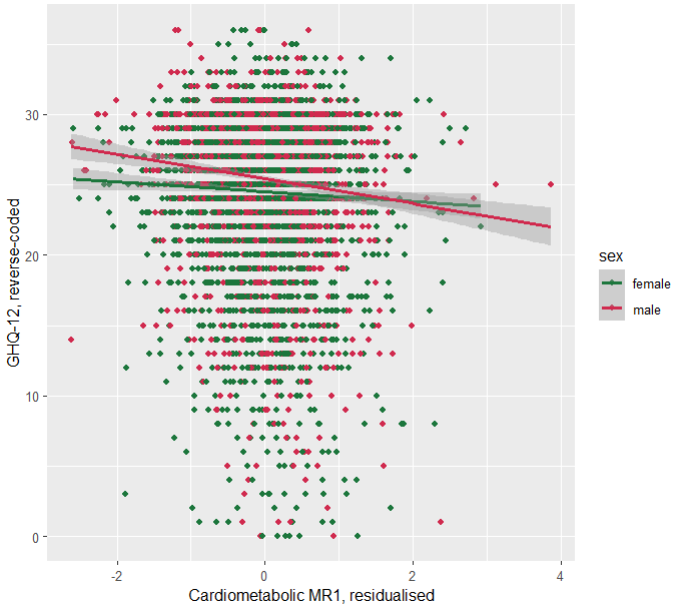


a. b.


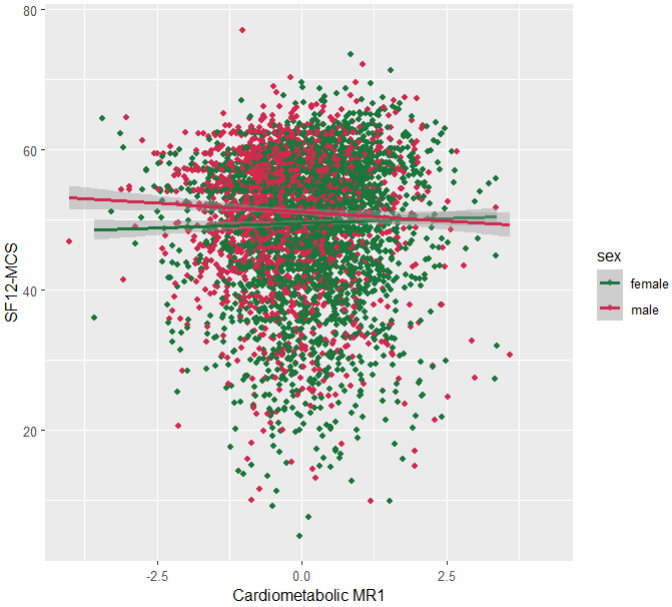

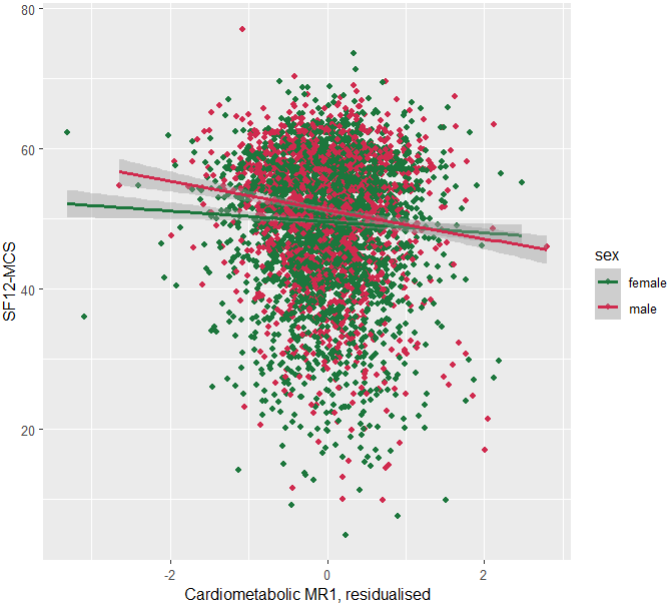


c. d.

Figure S2. Association of cardiometabolic MR1 with mental health. a-b: GHQ-12, c-d: SF12-MCS. Regression lines are plotted by sex. In a & c, raw values have been plotted, whereas in b & d, cardiometabolic MR1 has been residualised against the other terms in the respective model for each outcome (i.e. the other proteomic factors retained after backward selection, and their respective sex interactions, plus age, age squared, ethnicity and education, but not sex). Lower scores for GHQ-12 and SF12-MCS indicate more mental health symptoms.
